# Ocular Effects of Niacin: A Review of the Literature

**Published:** 2015

**Authors:** Daniela Domanico, Francesca Verboschi, Simona Altimari, Luigi Zompatori, Enzo Maria Vingolo

**Affiliations:** 1 Department of Medical–Surgical Sciences and Biotechnologies, U.O.S.D. Ophthalmology, Sapienza University of Rome, Polo Pontino, Terracina (LT), Italy; 2 Department of Ophthalmology, “San Giovanni Evangelista” Hospital, Tivoli (RM), Italy

**Keywords:** Cystoid Macular Edema, Niacin Maculopathy, Nicotinic Acid, Side Effects

## Abstract

Cystoid macular edema is a condition that involves the macula, caused by an accumulation of extracellular fluid in the macular region with secondary formation of multiple cystic spaces. This condition is provoked by a variety of pathological conditions such as intraocular inflammation, central or branch retinal vein occlusion, diabetic retinopathy and most commonly following cataract extraction, hereditary retinal dystrophies, and topical or systemic assumption of drugs. Niacin is a vitamin preparation usually used for the treatment of lipid disorders. The treatment with niacin, alone or in combination with other lipid-lowering agents, significantly reduces total mortality and coronary events and slows down the progression of and induces the regression of coronary atherosclerosis. Several cases of niacin-induced cystoid macular edema have been reported with different dosages.

## INTRODUCTION

Cystoid macular edema (CME) refers to swelling of the central part of the retina. The macula is responsible for detailed central vision. When the macula experiences swelling (edema), central vision is reduced. CME represents a common pathologic sequel of the retina and occurs in a variety of pathological conditions such as intraocular inflammation, central or branch retinal vein occlusion, diabetic retinopathy and most commonly following cataract extraction, hereditary retinal dystrophies, and topical or systemic assumption of drugs (1–2).

 Several cases of niacin induced CME have been reported (3–6). The aim of this review is to show the use of niacin and its correlation between administration of niacin and CME.

## CME: ETIOPATHOGENESIS

CME is a disorder that involves the central retina, caused by an accumulation of extracellular fluid in the macular region with secondary formation of multiple cystic spaces (7). Histological studies show that radially orientated cystoid spaces consisting of ophthalmoscopically clear fluid are often clinically detectable in the macula area. These cysts seem to be areas of the retina in which the cells have been displaced (1). It is the result of cystic accumulation of extracellular intraretinal fluid in the outer plexiform and inner nuclear layers of the retina (8).

The exact pathogenesis of CME remains uncertain. CME following disruption of the blood–retinal barrier (BRB) (9). When the BRB is damaged, fluid accumulates within the retina both intra- and extracellularly (10). Müller cells are thought to play an important role in acting as metabolic pumps that keep the macula dehydrated. However, intracellular fluid accumulation in the Müller cells may also occur in CME and further reduce macular retinal function. Vitreous traction may also play a part in the development of CME (11). Macular edema is the most frequent and severe complications of pars planitis, HLA-B27-associated acute anterior uveitis, sarcoidosis, birdshot retinochoroidopathy, Behcet’s syndrome, toxoplasmosis, Eales’ disease, idiopathic vitritis, Vogt-Koyanagi-Harada syndrome, and scleritis (12–17). CME can follow cataract surgery in the Irvine–Gass syndrome (18), because after cataract surgery, inflammatory mediators from the anterior chamber diffuse posteriorly into the vitreous, causing leakage from the retinal vasculature (19–20).

CME is the consequence of diabetic retinopathy (21). The edema in the macular area occurs secondary to an abnormal permeability of the capillaries surrounding the macula (failure of inner retinal blood barrier), and in turn to a failure in the outer retinal barrier (formed by the retinal pigmented epithelium) (22–24). Retinal vein obstructions (RVO) represent another cause of CME. Increased rigidity of a crossing artery because an atherosclerotic process has been suggested to cause compression of the underlying vein, provoking turbulent blood flow, endothelial damage, and thrombus formation (25). Likewise, a common vitreous adhesion at the obstruction site has also been reported, suggesting a possible role of vitreovascular traction in the etiology of some cases of BRVO (26–27). Atherosclerosis is a chronic low-grade inflammatory disorder and inflammation within the vascular wall contributes to the development of CME (28–29). Due to BRB breakdown secondary to damage at the tight junctions of endothelial cells, fluid diffusion from the occluded veins into the tissue can lead to CME (30). In addition, through such mechanisms, inflammatory responses and vascular dysfunction can all interact to cause retinal ischemia, which induces the expression of VEGF and others inflammatory agents (31), which participate in a complex chain of events that has yet to be fully defined (32–34).

## NIACIN: DEFINITION AND RULES

Niacin (nicotinic acid) is a vitamin preparation usually used for the treatment of lipid disorders (35). Niacin favorably affects apolipoprotein (apo) B–containing lipoproteins (very-low-density lipoprotein [VLDL], low-density lipoprotein [LDL], lipoprotein[a]) and increases apo A-I–containing lipoproteins (high-density lipoprotein [HDL]). There are new data on how niacin affects triglycerides (TG), vascular anti-inflammatory events, a particular niacin receptor in adipocytes and immune cells, and the characterization of a niacin transport system in liver and intestinal cells that is dependent on acidic pH, temperature, energy, Ca2+-calmodulin-mediated pathways, but transport is sodium independent (36–42). Niacin directly and noncompetitively inhibits hepatocyte diacylglycerol acyltransferase–2, a key enzyme in TG synthesis that results in accelerated intracellular hepatic apo B degradation. Niacin reduces apolipoprotein C-III levels by inhibition of peroxisome-proliferator-activated receptor gamma co-activator 1b (PGC-1b), allowing greater apoE-driven clearance of triglyceride-rich lipoproteins (43).

Niacin is available in a variety of formulations: immediate-release (IR), slow-release (SR), extended-release (ER) (44). Studies have demonstrated that ER provides lipid-modifying efficacy equivalent to that of IR niacin, but with less flushing, while avoiding the hepatotoxicity of other long-acting niacins (45–46). However, the use of niacin in patients with diabetes has been discouraged because high doses can worsen glycemic control (47). The discovery that niacin reduced free fatty acids (FFA) lead to suggestions that its primary effect was on peripheral lipolysis and that all other actions were secondary. This was reinforced by the discovery of the HM74⁄ GPR109A cyclic-G protein-coupled receptor in adipose tissue (48) and niacin is a specific ligand for this receptor which mediates the flushing response in dendritic cells and macrophages (49–50). Treatment with niacin, alone or in combination with other lipid-lowering agents, significantly reduces total mortality and coronary events and holds back the progression of and induces the regression of coronary atherosclerosis (37,51–52).

Niacin improves endothelial function (53–54) and reduces progression of carotid intima-media thickness (cIMT) (55) and also had this effect in trials on top of statin-optimised LDL-C when used at a low dose of 1 g ⁄ day in the Arterial Biology for the Investigation of the Treatment Effects of Reducing Cholesterol (ARBITER)-2 (& 3) trials (52,56).

## SYSTEMIC SIDE EFFECTS OF NIACIN

The problem with niacin is the high incidence of flushing (57). The flushing is cutaneous and usually restricted to the face and chest and is also associated with a burning sensation and often generalized pruritus. It lasts 20–30 min and habituates with exposure declining in both severity and frequency with time (58–59). 

Niacin-induced flushing appears to be due to the subcutaneous release of PGD2, which is mediated by niacin’s action as a pharmacologic ligand for the adipocyte and macrophage G protein–coupled nicotinic acid receptor GPR109A and appears to involve the formation of vasodilatory prostanoids (60–61). Epidermal Langerhans cells are essential mediators of the flushing response (62–65). Niacin also has adverse effects on some aspects of the metabolic syndrome: it is weight neutral and delivers a small blood pressure reduction (66) but it can increase glucose and HbA1c (67–69).

This effect is often temporary (70) but in 5–10% patients with diabetes it can raise HbA1c by 0.5–1.0% even if the alteration of hypoglycaemic therapy occurs (71). Clinical studies suggest that the niacin can raise the plasma levels of uric acid and reduce the glucose tolerance. Dry skin and cutaneous hyperpigmentation (acanthosis nigricans) are also commonly reported (72).

## NIACIN AND OCULAR EFFECTS

Many authors reported ocular side effect after therapy with niacin. Fraunfelder et al. (5) reported that 3 g or more per day of nicotinic acid, could cause blurred vision, eyelid edema, toxic amblyopia, proptosis, loss of eyelashes or eyebrow, superficial punctate keratitis and CME. Some cases of blurred vision were reported in the literature (73), and 18 cases were reported to the National Registry or the FDA spontaneous reporting system. In these cases, the average dose of niacin was 1.5–2 g per day, with a duration of therapy varying from 6 weeks to 1 year. Follow-up reported complete resolution of visual symptoms after discontinuing niacin. Dry eye was explained because this vitamin may be secreted and concentrated in human tears, thereby irritating an already dry eye. Niacin can cause ocular signs and symptoms reversible dose related, so if the patient wishes to continue this therapy, it may be feasible to reduce the dose of the drug (6). Cases of niacin related maculopathy there is a 10:1 male: female ratio. Most cases were in their third to fifth decade of life and were being treated with an average dose of 3–6 g of niacin per day. The etiology of niacin’s effect on the macula is unknown, A first hypothesis suggests that, in patients with vascular or inflammatory diseases, niacin induces the release of prostaglandins, causing the blood-retinal barrier compromise with extracellular accumulation of fluid in many cystic spaces. This theory is not entirely accepted for the absence of fluorescein leakage (74).

**Figure 1:**
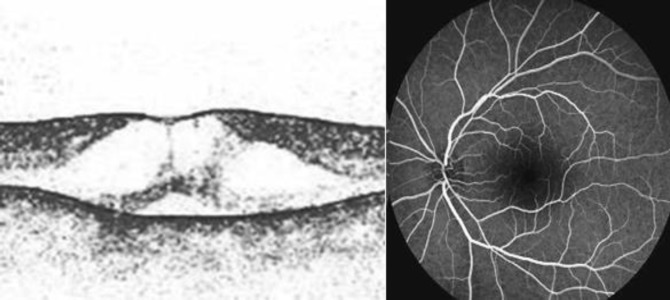
On the left, there is an image of Stratus OCT 2 weeks after the start of niacin treatment. On the right fluorescein angiography of the same patient after 3 weeks after the beginning of niacin treatment with the typical absence of leakage also in later phases

The second and the most reliable hypothesis supports that the niacin has a direct toxic effect on Müller cells, without disruption of the blood-retinal-barrier. Alterations in cellular metabolism cause intracellular fluid increase and swelling of these cells, with secondary formation of cysts between the glial spaces. After the discontinuation of niacin therapy, there is a complete regeneration of the Müller cells and their normal function (75–76). Reports from the literature (77) suggest that the onset of maculopathy ranges from 1 to 36 months after initiation of relatively high-dose therapy (3 g or more daily). There have also been reports of this condition with lower dosages (1.5 g daily) (3). This patient had increased the dosage of niacin to 3 g daily nearly 2 months before documentation of CME. In a previous case report a very low dose of niacin was administered (18 mg) and niacin maculopathy appears 4 weeks later with a complete resolution after discontinuation of drug, confirming the relationship between drug administration and macular edema appearance. In this patient, funduscopic examination showed bilateral macular edema, but no signs of diabetic or hypertensive retinopathy excluding the presence of other factors that may contribute to the capillary dysfunction associated with edema onset (78). At funduscopic examination, the foveomacular area had a peculiar aspect: the foveola takes on a bright yellow hue, similar to an exudate. Cysts primarily involve the foveal area, and they are smaller than those seen in post-surgical or inflammatory CME (3). FA and OCT are useful to confirm the diagnosis. FA showed the absence of fluorescein leakage or vascular alteration, whereas OCT confirms the presence of cystic hyporeflective spaces in the outer plexiform and inner nuclear layers. The cystoid spaces were more numerous and larger in the outer plexiform layer compared with the inner nuclear layer. Indeed previous studies reported that the fluor-angiographic pattern is atypical (79–81) (Fig. 1).

Gass described reversible bilateral CME with no angiographic fluorescein leakage in 3 patients taking doses of niacin greater than 1.5 g daily to treat hyperlipidemia (6). Although Gass described transmitted hyper-fluorescence with a cartwheel pattern in the fluorescein angiograms of his patients, later reports described typical fluorescence in similarly affected patients (3). In all these cases, symptoms resolved over 4 to 8 weeks following discontinuation of niacin. First-line treatment for CME of toxic origin, such as that induced by niacin, is the removal of the offending agent (77). In milder cases of maculopathy, symptoms have resolved within a few days of drug discontinuation.

## CONCLUSION

It was shown that the incidence of niacin maculopathy has been estimated to occur in 0.67% of patients being treated for hyperlipidemia. Studies have shown that even small doses of niacin cause CME. Patients with ocular symptoms such as blurred vision, decrease in visual acuity and metamorphopsia, should be immediately go to an ophthalmologist and stop the assumption of nicotinic acid before the onset of maculopathy. Niacin should be used with caution and under medical monitoring or periodic examination. Further studies would be desirable to investigate the safety dose, pathophysiologic mechanisms or predisposing risk factors and the possible interaction with others agents that may cause niacin maculopathy.

## DISCLOSURE

The authors report no conflicts of interest in this work.
